# Assessing the impact of heatwaves on emergency visits for major depression and suicidal ideation in youth with attention-deficit/hyperactivity disorder

**DOI:** 10.1371/journal.pmen.0000444

**Published:** 2025-10-29

**Authors:** Jennifer D. Runkle, Charlie Reed, Karla Weidner, Julia Rothschild, Tara Chandrasekhar, Margaret M. Sugg

**Affiliations:** 1 North Carolina Institute for Climate Studies, North Carolina State University, Asheville, North Carolina, United States of America; 2 Psychiatry & Behavioral Sciences, Duke University School of Medicine, Durham, North Carolina, United States of America; 3 Department of Geography and Planning, Appalachian State University, Boone, North Carolina, United States of America; UCL: University College London, UNITED KINGDOM OF GREAT BRITAIN AND NORTHERN IRELAND

## Abstract

Emerging evidence suggests that extreme heat can exacerbate mental health conditions. Yet little is known about its impact on children with attention-deficit/hyperactivity disorder (ADHD), a population at increased risk for emotional dysregulation and impulsivity. This study examines the association between heatwave exposure and emergency department visits for major depressive disorder (MDD), suicidal behavior (SUIC), and their co-occurrence among youth with ADHD. We conducted a retrospective matched-case study of 4,404 pediatric ED visits for MDD and/or SUIC in youth with ADHD in North Carolina from May to September, 2008–2021. Heatwave exposure was defined using the Excess Heat Factor and modeled as same-day, lagged (1–7 days), and cumulative (3-,5-, and 7-day) periods. Poisson mixed-effect regression models estimated relative risk (RR) and 95% confidence intervals (CI). Intersectional models further explored how risk varied by race, sex, ethnicity, and ADHD subtype. Heatwave exposure was significantly associated with increased ED visits for MDD (RR = 1.17, 95%CI: 1.01, 1.34) on the same day and overlapping MDD and SUIC (RR = 1.30, 95%CI: 1.02-1.65) on lag day one. Adolescents aged 12–17 showed heightened vulnerability across all outcomes. Cumulative exposure over 3-, 5-, and 7-day periods further elevated risks for MDD and suicidal behavior. Youth with the inattentive ADHD subtype had significantly greater odds of experiencing an overlapping MDD-suicidal event following heatwave exposure (RR = 2.70, 95%CI: 1.35-5.38). Intersectional analyses revealed that white females had the highest risk for suicide-related ED visits (RR = 1.21, 95%CI: 1.04-1.41). This study is the first to identify a link between heatwave exposure and mental health crises in youth with ADHD, emphasizing the need for targeted interventions. Findings highlight the importance of integrating climate resilience strategies into pediatric mental health care, particularly for high-risk subgroups.

## Introduction

Attention deficit hyperactivity disorder (ADHD) is a childhood-onset neurodevelopmental disorder marked by inattention, hyperactivity, and impulsivity [1,2]. Diagnosis and treatment varies globally, with increasing prevalence in the U.S., where 10.2% of all children between the ages of 3–17 are affected [3]. Boys are diagnosed at a higher rate than girls, as female presentations have been historically underrecognized [4,5]. ADHD diagnosis trends have shifted; diagnoses are now more common in Black compared to White youth, followed by Hispanic and Asian populations [6].

Approximately 77% of children with ADHD in the U.S. receive treatment, which is often initiated due to academic and behavioral challenges [7]. Medication is a common form of treatment, with nearly two-thirds of children with ADHD taking stimulants, such as amphetamines and methylphenidate [7]. These medications increase extracellular dopamine and norepinephrine, improving attention but also raising core body temperature by increasing heat rate, blood pressure, and dehydration risk [8–12]. Case studies and clinical reviews suggest that stimulant use may elevate heat-related illness (HRI) in children taking ADHD medications [8,13]. Given rising global temperatures, children with ADHD may be especially susceptible to extreme heat-related psychiatric events, particularly those with comorbid mood disorders or suicidal behaviors [14]. The presence of comorbidities may elevate vulnerability to heat both through direct mechanisms (e.g., treatment with psychotropic medications that affect thermoregulation) and indirect mechanisms (e.g., broader psychiatric burden leading to increased susceptibility to stressors such as heat exposure) [14].

The Southeastern U.S. is experiencing more frequent extreme heat days, with projections resembling present-day Florida’s tropical climate [15,16]. The Fourth National Climate Assessment confirms increasing heat extremes since the 1960s, with hazards heat-humidity combinations (wet bulb temperature ≥ 35°C) occurring faster than expected, particularly in coastal subtropical locations [15]. These trends underscore the urgency of identifying heat-sensitive populations, such as youth with ADHD, who may experience disproportionate health risks from rising temperatures.

Children and adolescents with ADHD face significantly higher risks of major depression and suicide, which may be partly explained by underlying neurobiological differences [17–24]. Research also suggests that girls and those with inattentive (ADHD-I) or combined (ADHD-C) subtypes may have a higher predisposition to comorbid depression and lethal suicide attempts [21,22,25,26]. These elevated risks have been linked to ADHD-related neurobiological changes and to the effects of stimulant medication or emotional regulation. Another mechanism involves the disorder’s strong association with impulsive decision-making and risk-taking behaviors, which peak in adolescence [19,27–30]. Adolescents with ADHD often demonstrate heightened impulsivity and risk-taking behaviors, driven more by impaired decision-making processes than by true risk-seeking tendencies, yet the mental health trajectories and factors influencing these behaviors remain understudied [31]. Extreme heat has been linked to increased aggression and impulsivity, raising concerns that such environmental stressors may compound the psychiatric vulnerabilities already faced by youth with ADHD [32].

Children with ADHD may experience heightened vulnerability to extreme heat, not only due to physiological and behavioral mechanisms, but also because of intersecting social identities that shape their lived environments. Families in under-resourced or rural communities may encounter barriers to timely health care and mental health support while structural factors such as housing quality and neighborhood environments (e.g., tree canopy) can influence both extreme heat exposure and adaptive capacity [33–37]. Sociocultural factors, such as stigma surrounding mental health, gendered help-seeking or symptom recognition and racialized disparities in diagnosis, treatment, and access to services can further compound heat risks [38–41]. Therefore, we applied an intersectional framework to capture how overlapping identities and social determinants jointly shape children’s vulnerability to heatwaves [42].

Despite growing evidence on the intersection of ADHD, mental health, and heat exposure, no population-based research has examined the connection between extreme heat and major depression or suicidal emergencies in children with ADHD. This exploratory study aims to examine the association between heatwave exposure and emergency department visits for suicide or major depression in individuals aged 5–24 years with ADHD. A secondary objective is to examine differences in risk across ADHD subtypes and how intersecting social identities influence heat vulnerability. We chose to focus specifically on major depressive disorder and suicidal emergencies because these outcomes reflect clinically severe psychiatric events and are rising in prevalence among youth with ADHD. Our approach was also informed by mounting evidence linking extreme heat to affective dysregulation and acute mental health outcomes in other populations, underscoring the need to investigate whether children with ADHD face similar vulnerabilities. By addressing this critical gap, our study generates hypotheses about the pathways through which extreme heat may precipitate acute psychiatric events in children with ADHD, providing an early evidence base to inform the development of future targeted interventions.

## Methods

This study extends the Differential Susceptibility Model to examine how youth with ADHD may be uniquely affected by extreme heat. Core ADHD traits (i.e., impulsivity, emotional reactivity, and sensory sensitivity) align with established markers of environmental susceptibility. Research indicates impulsivity and emotional reactivity shape responses to environmental influences, though studies have largely focused on social contexts (e.g., parenting, maternal stress) [43,44]. The model’s “plasticity gradient” suggests variability in heat sensitivity among youth with ADHD [43,45,46]. This framework guides our exploration of how ADHD subtypes and intersecting social identities may influence heat-related mental health risks.

### Study population

We obtained daily emergency department (ED) visits for all pediatric patients (ages 5–24 years) in North Carolina (NC) between January 1, 2008, and October 1, 2021, accessed through the University of North Carolina Cecil G. Sheps Center for Health Services Research. This statewide database, established under the Medical Data Act of 1995, captures discharge records from all hospitals in North Carolina. Records include patient demographics, diagnostic and procedure codes, payer information, admission date and source, discharge status, and length of stay [47]. For this study, we restricted the sample to ED visits for pediatric cases with an existing ADHD diagnosis (ICD-9:314.01; ICD-10: F90.9).

The primary health outcomes included ED visits with a primary or secondary diagnosis of: (1) major depressive disorder (MDD); (2) suicidal behavior (SUIC); or (3) co-occurring major depressive disorder and suicidal behavior (overlap) (S1 Table). Given the heightened suicide risk in youth with ADHD and comorbid depression, we included the overlap category [48]. To address coding uncertainty and ensure sufficient sample size for these rare outcomes, we included both primary and secondary diagnosis codes, a common and well-established approach in studies using hospital administrative data [49–52]. Data were de-identified prior to access by the research team. The NC State University institutional review board approved this secondary data analysis and waived the requirement for written informed consent (protocol approval #27048).

Demographic variables extracted from ED records included age, sex (i.e., physician-report of male, female), race (White, Black, and Other People of Color (OPC)), ethnicity (Hispanic, non-Hispanic) and county of residence. The OPC category was used to enhance statistical power by ensuring sufficient sample sizes within each group, while acknowledging racial/ethnic disparities in ADHD diagnosis and treatment [53]. This approach aligns with previous epidemiologic studies that have used similar categorizations to examine racial and ethnic health disparities through an intersectional lens [54]. ADHD subtypes (hyperactive, inattentive, combined, and unspecified) were identified using diagnosis codes (S1 Table), though ICD-9 lacked codes for combined and unspecified subtypes, potentially limiting precision.

This study applied an intersectional lens to examine how race, sex, and ADHD subtype may jointly shape vulnerability to heatwaves [55,56]. ADHD prevalence, symptoms, and treatment differ across these dimensions – for example, male children and non-Hispanic white children are more likely to receive stimulant medications [4,6,53]. Because stimulant use may influence thermoregulation, such differences could plausibly affect heat sensitivity. By constructing intersecting identity groups (e.g., sex by race, sex by ADHD subtype, race by ADHD subtype), we aimed to generate hypotheses about whether overlapping social and clinical identities reveal disparities that would be underestimated if examined in isolation [53,57].

### Extreme heat data

Prior research indicates that sustained heatwaves exert more severe and compounded effects on human health by straining thermoregulation, mental well-being, and healthcare systems, compared to isolated hot days measured by short-term fluctuations in daily ambient temperature [58]. Heatwaves are a period of sustained and cumulative heat stress – three consecutive days or longer – that have more consequential physiological effects than a single day of high temperature and pose greater risk for younger populations [59–61]. To measure heatwave exposure, daily average temperatures (Tavg) for each NC county were obtained from NOAA’s nClimGrid product [62]. Heatwaves were defined using the Excess Heat Factor (EHF), which captures a climatology for each county and the acclimatization of the population within a 30-day period (i.e., intensity and duration of extreme heat relative to local climate norms) [63]. This approach is particularly advantageous for health impact studies as it captures both absolute heat stress and relative changes affecting physiological adaptation [64]. EHF combines two metrics: 1) *EHF Significance Index* (EHFsig), comparing the current 3-day mean temperature to the long-term 95th percentile threshold, and 2) *EHF Acclimatisation Index* (EHFaccl), accessing deviation from the preceding 30-day average. A heatwave day was defined as EHF > 0 and coded as a binary variable.

### Statistical analysis

We calculated temperature averages on heatwave days and non-heatwave days across the state and for each individual county in NC. Descriptive statistics characterized pediatric ADHD-related ED visits, stratified by heatwave and non-heatwave days.

We employed a matched case study design, following an established methodology in climate health research [65–68]. Heatwave days were designated as case days each matched with three reference non-heatwave days that had to meet the following criteria: (a) occur in the same county as the case day, (b) occur within the same month as the case day, (c) do not coincide with or fall within 3 days of a heatwave event, and (d) do not occur in the same year as the case [65].

ED visits for MDD, SUIC, and overlap were aggregated separately by day and county. Prolonged exposure to extreme heat can lead to accumulated physiological and behavioral stress, potentially resulting in delayed health impacts. Following prior research studies, to capture both immediate and lagged responses, we examined same-day (lag 0) and single-day lags of 1–7 days [50,69–71]. Additionally, we assessed cumulative exposure effects over 3-, 5-, and 7-day intervals, which are commonly applied in climate-health studies to balance physiological plausibility with epidemiological practice [50,70,72]. Whereas single-day lags capture short-term fluctuations in exposure-response, multi-day lags provide insight into the cumulative burden of sustained heat exposure [59].

Poisson mixed-effects regression models estimated the association between heatwaves and each outcome, adjusting for population size using an offset variable. Adaptive quadrature was applied to improve computational efficiency and accuracy when integrating over random effects [73]. In cases where adaptive quadrature failed to produce reliable confidence intervals for effect estimates, we applied the Laplace approximation instead [74]. The model included a county-specific random intercept and fixed effects for day of week, year, and a binary indicator variable to control for warm season trends (May through September). In our study, non-heatwave control days were restricted to the same county and calendar month as the case day but excluded from the same year to reduce potential overlap and contamination with heatwave exposure. This approach also increases the pool of eligible non-heatwave days, ensuring more robust matching while still controlling for seasonality, long-term temporal trends, and county-specific factors.

The models estimated the rate ratio (RR) and 95% confidence intervals of ED visits on heatwave days compared to non-heatwave days. We analyzed single-day and multi-day lags individually, in separate models. Next, we examined effect modification by sex, race, ADHD subtype, and age group. To further explore intersectional risks, we conducted additional models examining: (1) race and sex; (2) race and ADHD subtype; and (3) sex and ADHD subtype, allowing for overlapping social categories to better understand how multiple identities contribute to heatwave-related health risks. Because running multiple tests for our subgroup intersectional analysis may have increased the likelihood of a Type I error, we applied the Benjamini–Hochberg False Discovery Rate (FDR) procedure to control the expected proportion of false positives, reducing the likelihood of spurious findings [75].

To assess the robustness of our findings, we conducted sensitivity analyses by (1) restricting the data to the warm season only and (2) examining ADHD-related ED visits listed as either a primary or secondary diagnosis, rather than cases where ADHD co-occurred with MDD or suicidal behavior. All analyses were performed using SAS software, Version 9.4 [76]. Two-sided statistical tests were used, and 95% confidence intervals were calculated to evaluate the precision of estimates.

## Results

From a total of 356,072 ED visits of youth with ADHD, 4,404 visits met the inclusion criteria: 1,029 (23%) admitted for MDD and 3,712 (84%) for SUIC (Table 1). Of these, 337 individuals (8%) were admitted for both conditions, termed “overlap”. Half of the sample (50%) were between 12 and 17 years old; 60% were white, and 55% had an unspecified ADHD subtype. While males comprised the majority (64%) of the overall ADHD sample, females accounted for a larger proportion of visits for MDD-related visits (53%). The hyperactive ADHD subtype was diagnosed in 1,309 individuals (30%), with a higher prevalence among those admitted for suicidal behavior (32%) compared to MDD (19%).

**Table 1:** Demographic characteristics of 4,404 youth with ADHD who visited the ED for each outcome stratified by heatwave (cases) and non-heatwave days (reference).

	MDD		SUIC		Overlap	
	HW-day	NonHW-day	HW-day	NonHW-day	HW-day	NonHW-day
	Freq (col%)	Freq (col%)	Freq (col%)	Freq (col%)	Freq (col%)	Freq (col%)
**Number of Days**	34,280 (NA)	102,840 (NA)	35,764 (NA)	107,292 (NA)	35,764 (NA)	107,292 (NA)
**Number of ED Admissions**	286 (NA)	743 (NA)	933 (NA)	2,779 (NA)	100 (NA)	237 (NA)
**Sex**						
Male	138 (48)	350 (47)	527 (56)	1624 (58)	40 (40)	109 (46)
Female	148 (52)	393 (53)	406 (44)	1155 (42)	60 (60)	128 (54)
**Race**						
White	193 (69)	477 (65)	545 (60)	1659 (61)	69 (71)	160 (70)
Black	70 (25)	210 (29)	259 (29)	774 (29)	24 (25)	62 (27)
Other	15 (6)	43 (6)	102 (11)	269 (10)	4 (4)	8 (3)
**Ethnicity**						
Hispanic	10 (4)	37 (5)	35 (4)	97 (4)	3 (3)	5 (2)
Not Hispanic	261 (96)	684 (95)	793 (96)	2425 (96)	91 (97)	226 (98)
**Age**						
5–11	13 (5)	36 (5)	132 (14)	389 (14)	4 (4)	6 (3)
12–17	148 (52)	390 (52)	468 (50)	1355 (49)	54 (54)	121 (51)
18–24	125 (44)	317 (43)	333 (36)	1035 (37)	42 (42)	110 (46)
**ADHD Subtype**						
Hyperactive	45 (16)	150 (20)	273 (29)	901 (32)	10 (10)	50 (21)
Inattentive	29 (10)	61 (8)	53 (6)	190 (7)	16 (16)	19 (8)
Combined	47 (16)	105 (14)	69 (7)	212 (8)	14 (14)	27 (11)
Unspecified	167 (58)	432 (58)	539 (58)	1479 (53)	60 (60)	141 (59)

HW: Heatwave; Column percentages for number and days and number of cases did not apply. MDD = Major Depressive Disorder; SUIC: Suicidal behavior; Overlap: Co-occurring Major Depressive Disorder and Suicidal behavior

The average temperature on heatwave days was notably higher than non-heatwave days (Table 2). The maximum temperature during a heatwave event was 40.8 °C (105.4 °F) compared to 36.9 °C (98.4 °F) on a non-heatwave day. The south-central region of the state was generally the hottest, with seven counties averaging above 28°C (82 °F) on heatwave days and experiencing at least one heatwave day above 40 °C (104 °F) (S2 Table).

**Table 2:** Temperature averages, minimum, and maximums on heatwave and non-heatwave days across North Carolina (2008-2021). Heatwave days were identified using the excess heat factor for each day in each county.

	Average Statewide Temperature (°C)			
	Mean (SD)	Median (IQR)	Minimum	Maximum
Heatwave Days	27.0 (2.3)	27.6 (2.3)	9.2	40.8
Non-heatwave Days	14.5 (8.2)	15.2 (13.7)	-22.8	36.9

The results showed significant associations between heatwave exposure and the outcomes of interest (Table 3). ED visits for MDD increased on the same day and the day after a heatwave (i.e., Lag1) compared to matched non-heatwave days. Similarly, the risk of overlapping MDD and SUIC increased one day after a heatwave. Cumulative effects over 3-day, 5-day, and 7-day lag periods showed elevated risks for MDD and SUIC, with the strongest effects observed over the 3-day period. However, these cumulative effects were attenuated for overlapping MDD and SUIC compared to same-day or single-day lags.

**Table 3:** Relative risk (RR) and 95% confidence intervals (95%CI) derived from Poisson mixed-effect regression from same-day, lagged, cumulative, and covariate models for each outcome (1) major depressive disorder (MDD); (2) suicidal behavior (SUIC); or (3) co-occurring or overlapping major depressive disorder and suicidal behavior (overlap).

Variable	MDD			Suicide			Overlap		
	RR	95% CI	p	RR	95% CI	p	RR	95% CI	p
Same Day	1.17	(1.01, 1.34)	0.03	1.03	(0.96, 1.12)	0.08	1.27	(1.00, 1.62)	0.05
Lag 1	1.17	(1.02, 1.35)	0.02	1.04	(0.96, 1.12)	0.06	1.30	(1.02, 1.65)	0.03
Lag 2	1.14	(0.99, 1.32)	0.07	0.99	(0.92, 1.08)	0.44	1.24	(0.97, 1.58)	0.44
Lag 3	1.09	(0.95, 1.26)	0.23	0.99	(0.92, 1.07)	0.47	1.15	(0.90, 1.48)	0.47
Lag 4	1.02	(0.88, 1.19)	0.76	0.97	(0.90, 1.05)	0.86	1.07	(0.83, 1.38)	0.86
Lag 5	1.00	(0.86, 1.16)	0.95	0.98	(0.91, 1.06)	0.65	0.98	(0.91, 1.06)	0.65
Lag 6	0.99	(0.85, 1.15)	0.88	1.02	(0.94, 1.10)	0.18	1.02	(0.94, 1.10)	0.18
Lag 7	0.97	(0.83, 1.12)	0.65	1.01	(0.93, 1.09)	0.23	1.03	(0.80, 1.34)	0.22
3-day cumulative	1.14	(1.07, 1.23)	0.00	1.05	(1.01, 1.09)	0.02	1.15	(0.90, 1.48)	0.46
5-day cumulative	1.10	(1.04, 1.17)	0.00	1.04	(1.01, 1.08)	0.02	1.05	(0.89, 1.25)	0.44
7-day cumulative	1.07	(1.02, 1.13)	0.01	1.04	(1.01, 1.07)	0.00	1.03	(0.80, 1.34)	0.20
Male	1.21	(0.99, 1.48)	0.07	1.03	(0.93, 1.14)	0.60	1.17	(0.81, 1.71)	0.60
Female	1.13	(0.93, 1.38)	0.21	1.14	(1.01, 1.28)	0.04	1.34	(0.98, 1.84)	0.08
Age 5–11	1.02	(0.53, 1.97)	0.95	1.02	(0.83, 1.26)	0.84	1.89	(0.51, 7.00)	0.84
Age 12–17	1.20	(0.99, 1.46)	0.07	1.19	(1.06, 1.33)	0.00	1.46	(1.05, 2.04)	0.00
Age 18–25	1.15	(0.92, 1.42)	0.22	0.96	(0.84, 1.09)	0.55	1.04	(0.72, 1.51)	0.55
White	1.26	(1.06, 1.50)	0.01	1.07	(0.97, 1.18)	0.19	1.27	(0.95, 1.69)	0.19
Black	0.98	(0.74, 1.29)	0.87	1.15	(0.99, 1.33)	0.07	1.03	(0.80, 1.34)	0.07
Other	0.87	(0.46, 1.61)	0.65	0.87	(0.46, 1.61	0.65	1.33	(0.38, 4.72)	0.65
Hyperactive	0.70	(0.49, 1.00)	0.05	0.94	(0.81, 1.09)	0.43	0.47	(0.23, 0.95)	0.44
Inattentive	1.47	(0.93, 2.31)	0.10	0.88	(0.64, 1.22)	0.44	2.70	(1.35, 5.38)	0.44
Combined	1.48	(1.04, 2.10)	0.03	1.03	–	–	1.34	–	–
Unspecified	1.24	(1.03, 1.49)	<0.0	1.18	–	–	1.37	(1.00, 1.86)	0.05
Hispanic	0.89	(0.44, 1.83)	0.76	1.06	(0.71, 1.58)	0.76	1.87	(0.43, 8.14)	0.76
Non-Hispanic	1.18	(1.02, 1.37)	0.02	1.05	(0.97, 1.14)	0.31	1.22	(0.95, 1.57)	0.11

*Same-day effects were analyzed by covariates to assess potential effect modification by demographic factors.

- Indicates that the model could not compute RR or 95% confidence interval.

Table 3 illustrates the role of effect modifiers for MDD, SUIC, and overlapping MDD and SUIC respectively. Females with ADHD were more likely to present to the ED for SUIC on heatwave compared to non-heatwave days (RR: 1.14, 95%: 1.01, 1.28). While not statistically significant, males showed a higher risk of MDD-related ED visits (RR: 1.21, 95%: 0.99, 1.48), while females had a greater risk of overlapping diagnoses (RR: 1.34, 95%: 0.98, 1.84). Adolescents (12–17 years) with ADHD were at higher risk for SUIC (RR: 1.19, 95%: 1.06, 1.33) and overlapping diagnoses (RR: 1.46, 95%: 1.05, 2.04). White youth were more likely to visit the ED for MDD on heatwave compared to non-heatwave days (RR: 1.26, 95%: 1.06, 1.50). Regarding ADHD subtypes, youth with combined or unspecified ADHD subtypes had an increased risk of MDD-related ED visit on heatwave days (RR: 1.24, 95%: 1.03, 1.49). The inattentive subtype was associated with a 2.7-fold increase in risk for overlapping diagnoses (95%: 1.35, 5.38). Non-Hispanic youth were more likely to present with MDD on heatwave compared to non-heatwave days (RR: 1.18, 95%: 1.02, 1.37).

The intersectional models revealed more nuanced patterns of risks (Table 4, S1-S3 Figs). After adjusting for multiple tests, white children with the unspecified ADHD subtype were more likely to visit the ED for MDD (RR: 1.36, 95%: 1.08-1.71), as were males with the unspecified subtype (RR: 1.49, 95%: 1.15-1.92) and females with the combined subtype (RR: 2.06, 95%: 1.27-3.33). White male youth were more likely to be admitted for MDD (RR: 1.29, 95%: 1.01, 1.66), however the Benjamini-Hochberg test suggests that this is likely a false positive. White female youth were much more likely to be admitted for suicide (RR: 1.21, 95%: 1.04, 1.41) on heat wave verses non-heatwave days. After FDR correction, other youth of color with undefined subtype exhibited a higher risk for a suicide-related ED visit and White youth with inattentive ADHD were 4.4 times more likely to present with both MDD and suicidal behavior (95%:1.83, 10.43). We also observed higher risk of both MDD and suicidal behavior among non-Hispanic youth with the Inattentive subtype of ADHD (RR: 2.26, 95%: 1.10-4.62).

**Table 4:** Relative risk (RR) and 95% confidence intervals (95%CI) derived from Poisson mixed-effect regression across intersecting demographic characteristics for each outcome. False Discovery Rate used to correct p-values for inflated Type I error often associated with multiple hypothesis testing.

Intersection	MDD				SUIC				Overlap			
	RR	95% LB	p	FDR	RR	95% CI	p	FDR	RR	95% CI	p	FDR
White x Male	1.29	(1.01, 1.66)	0.04	0.13	0.98	(0.85, 1.12)	0.72	0.97	1.10	(0.70, 1.72)	0.73	0.97
White x Female	1.23	(0.97, 1.56)	0.09	0.25	1.21	(1.04, 1.41)	0.01	0.05	1.42	(0.96, 2.09)	0.80	0.05
Black x Male	1.07	(0.71, 1.60)	0.75	0.97	1.07	(0.71, 1.60)	0.75	0.97	1.20	–	–	–
Black x Female	0.89	(0.60, 1.32)	0.57	0.90	0.89	(0.60, 1.32)	0.57	0.91	1.15	–	–	–
Other x Male	0.95	(0.39, 2.32)	0.92	0.97	1.07	(0.90, 1.27)	0.43	0.74	1.79	(0.23, 13.75)	0.96	0.97
Other x Female	0.79	(0.33, 1.91)	0.61	0.90	1.06	(0.87, 1.29)	0.56	0.91	1.18	(0.23, 6.10)	0.91	0.96
White x Sub H.	0.83	(0.55, 1.27)	0.39	0.64	1.01	(0.83, 1.22)	0.96	0.75	0.57	(0.26, 1.27)	0.85	0.97
White x Sub I.	1.72	(1.01, 2.93)	0.04	0.13	0.96	(0.63, 1.46)	0.85	0.90	4.37	(1.83, 10.43)	0.00	<0.01
White x Sub C.	1.37	(0.90, 2.10)	0.14	0.34	1.03	(0.71, 1.50)	0.88	0.97	1.27	(0.55, 2.91)	0.87	0.97
White x Sub U.	1.36	(1.08, 1.71)	0.01	0.05	1.12	–	–	–	1.23	–	–	–
Black x Sub H.	0.42	(0.20, 0.90)	0.03	0.12	0.42	(0.20, 0.90)	0.02	0.03	0.14	(0.02, 1.18)	0.24	0.36
Black x Sub I.	1.03	(0.34, 3.14)	0.97	0.97	1.03	(0.34, 3.14)	0.97	0.96	1.23	(0.25, 6.01)	0.53	0.96
Black x Sub C.	1.66	(0.80, 3.43)	0.17	0.36	1.66	(0.80, 3.43)	0.17	0.17	1.46	(0.43, 4.96)	0.23	0.49
Black x Sub U.	1.04	–	–	–	1.04	–	–	–	1.55	(0.85, 2.83)	0.17	0.97
Other x Sub H.	0.87	(0.23, 3.28)	0.83	0.97	0.87	(0.68, 1.10)	0.25	0.25	0.65	(0.03, 12.52)	0.98	0.97
Other x Sub I.	0.56	(0.04, 7.13)	0.65	0.92	0.76	(0.43, 1.33)	0.33	0.33	0.02	–	–	–
Other x Sub C.	3.18	(0.55, 18.29)	0.19	0.39	1.04	–	–	–	–	–	–	–
Other x Sub U.	0.63	(0.26, 1.53)	0.31	0.53	1.25	(1.05, 1.47)	0.01	0.01	1.00	(0.20, 4.99)	0.97	0.97
Hispanic x Male	0.79	(0.22, 2.83)	0.72	0.97	1.02	(0.61, 1.72)	0.72	0.97	–	–	–	–
Hispanic x Female	0.95	(0.40, 2.26)	0.91	0.97	1.11	(0.60, 2.06)	0.91	0.97	2.83	(0.56, 14.47)	0.55	0.72
Non-Hispanic x Male	1.25	(1.02, 1.55)	0.04	0.12	0.99	(0.89, 1.11)	0.34	0.96	1.22	(0.83, 1.79)	0.23	0.39
Non-Hispanic x Female	1.12	(0.91, 1.38)	0.28	0.50	1.14	(1.00, 1.29)	0.05	0.09	1.21	–	–	–
Hispanic x Sub H.	–	–	–	–	0.80	(0.33, 1.90)	0.28	0.55	–	–	–	–
Hispanic x Sub I.	1.30	(0.13, 12.66)	0.82	0.97	–	–	–	–	0.02	–	–	–
Hispanic x Sub C.	2.08	(0.12, 36.95)	0.62	0.90	3.11	(0.68, 14.28)	0.82	0.97	–	–	–	–
Hispanic x Sub U.	1.06	(0.47, 2.40)	0.88	0.97	1.33	–	–	–	2.83	(0.56, 14.47)	0.67	0.90
Non-Hispanic x Sub H.	0.77	(0.53, 1.12)	0.17	0.36	0.93	(0.79, 1.11)	0.83	0.94	0.43	(0.20, 0.92)	0.04	0.08
Non-Hispanic x Sub I.	1.345	–	–	–	0.97	(0.68, 1.39)	0.80	0.96	2.26	(1.10, 4.62)	0.01	0.04
Non-Hispanic x Sub C.	1.48	(1.03, 2.13)	0.03	0.13	0.96	–	–	–	1.42	–	–	–
Non-Hispanic x Sub U.	1.24	(1.02, 1.50)	0.03	0.13	1.12	(1.01, 1.24)	0.03	0.10	1.31	(0.95, 1.81)	0.14	0.27
Male x Sub H.	0.73	(0.44, 1.22)	0.23	0.44	0.98	(0.81, 1.19)	0.85	0.97	0.77	(0.31, 1.92)	0.17	0.29
Male x Sub I.	0.95	(0.41, 2.24)	0.91	0.97	0.55	(0.33, 0.92)	0.02	0.10	1.65	(0.42, 6.47)	0.85	0.97
Male x Sub C.	1.01	(0.59, 1.74)	0.97	0.97	0.96	(0.66, 1.39)	0.83	0.97	0.34	(0.08, 1.49)	0.83	0.94
Male x Sub U.	1.49	(1.15, 1.92)	<0.01	<0.01	1.13	–	–	–	1.52	–	–	–
Female x Sub H.	0.67	(0.42, 1.10)	0.11	0.28	0.88	(0.69, 1.13)	0.32	0.60	0.24	(0.07, 0.82)	0.02	0.08
Female x Sub I.	1.80	(1.04, 3.13)	0.03	0.12	1.27	(0.84, 1.93)	0.26	0.55	3.00	(1.33, 6.74)	0.02	0.07
Female x Sub C.	2.06	(1.27, 3.33)	0.00	0.02	1.12	–	–	–	2.77	–	–	–
Female x Sub U.	1.03	–	–	–	1.12	(0.73, 1.72)	0.77	0.93	1.26	–	–	–

Sub H. = Hypertensive subtype. Sub I. = Inattentive subtype. Sub C. = Combination subtype. Sub U. = Unknown subtype. - Indicates that model could not compute RR or 95% confidence interval. MDD = Major Depressive Disorder; SUIC: Suicidal behavior; Overlap: Co-occurring Major Depressive Disorder and Suicidal behavior.

FDR: False Discovery Rate.

Sensitivity analysis for same day, lagged, or cumulative heatwave exposure in the warm season demonstrated slightly stronger associations with suicide-related ED visits (S3 Table); while overall results remained consistent with the annual results. An additional analysis limited to ED visits where ADHD was the primary or secondary diagnosis showed no increased risk on heatwave day (S4 Table). In some cases, cumulative heatwave exposure appeared to have a protective effect, particularly among White males and those with inattentive ADHD.

## Discussion

To our knowledge, this is the first study to examine the relationship between heatwaves and emergency department visits for major depressive disorders or suicidal behavior in pediatric patients with ADHD. Our findings indicate that children with ADHD were significantly more likely to visit the ED for major depressive or an overlapping depressive and suicidal episode on the same day or the day following a heatwave, with adolescents (12–17 years) at highest risk across all age groups. Cumulative heat exposure over 3-, 5-, and 7-day periods was also associated with increased risks for MDD, suicidal behavior, and their overlap. These results highlight the need for proactive, community-based strategies that leverage the strengths of children with ADHD, such as creativity, resilience, and problem-solving abilities, to build adaptive coping mechanisms and support mental well-being during extreme heat events.

Our findings are consistent with the existing literature linking ambient heat and adverse mental health outcomes [49,77–79]. A meta-analysis of 53 studies observed modest, but significant associations between heat exposure and mood disorders (RR = 1.011 [1.003-1.018]), as well as suicides and self-harm (RR = 1.012 [1.003-1.021]), but these studies focused exclusively on adults [80]. Limited research has explored the mental health effects of heat on children and adolescents, with only a few studies addressing this gap [81–84]. Notably, one case-crossover study found that higher minimum temperatures increased ED visits for intentional injury among 15–19 year olds (OR=1.34 [1.15-1.57]) and 20–25 year olds (OR=1.39 [1.20-1.61]), though broader studies on pediatric mental health have yielded mixed results [81–83].

Children with ADHD face heightened risk for psychiatric comorbidities, including major depression and suicide attempts, persisting into adolescence [21,22]. Emerging evidence links increasing ambient temperatures to increased suicide, depression, and impulsivity, particularly in adults, raising concerns for vulnerable pediatric populations [85]. Additionally, certain antidepressants and psychotropic medications, commonly prescribed for ADHD, can impair thermoregulation and increase susceptibility to heat-related illness [86]. Our results highlight a pronounced vulnerability of adolescents (12–17 years) that may reflect heightened emotional reactivity during this developmental stage.

We observed important sex-based differences. During heatwaves, White females with ADHD were more likely to present to the ED for suicidal episodes. ADHD subtype also modified risks: females with combined ADHD faced higher MDD risk and, females with inattentive subtype were nearly three times more likely to present with an overlapping diagnosis. Though these sex-based differences align with a previous systematic review showing an increased risk of suicide attempts in females, white females, and females with ADHD, the underlying mechanism is not well understood [25,87–91]. Women and girls with ADHD are more prone to internalizing symptoms, such as rumination, negative thoughts, anxiety, and depression [92]. These symptoms, which are also more common among people with a history of suicide attempts, may mediate the relationship between inattentiveness and other ADHD characteristics, extreme heat exposure, and self-harm [89,91]. Our analysis of co-presenting MDD and suicidal behavior tangentially supports this hypothesis. Females with the inattentive subtype of ADHD were at significantly greater risk of admission; and white females were positively – though insignificantly – associated with admission for MDD and suicidal behavior following heatwave exposure. Self-injury and suicidal behaviors rarely exist from single vulnerabilities; rather, they are embedded within independent family, peer, and socio-cultural systems that reinforce risk over time. Maladaptive family responses (e.g., coercion or emotional lability) and peer influence (e.g., social contagion, positive reinforcement of self-harm within certain groups) may help explain the sex- and subtype-specific patterns we observed, suggesting that sociocultural contexts may interact with neurobiological vulnerabilities to shape heat-related risks [93]. These potential sociocultural mechanisms highlight potential prevention targets that include parental monitoring and early intervention before risky peer affiliations that warrant further investigation.

Our findings challenge the hypothesis that impulsivity is the primary driver of heat-related mental health risks among children with ADHD. We did not observe an association between heatwaves and MDD or suicidal behavior in those with hyperactive subtype. Instead, significant risk for MDD emerged among children with inattentive subtype, characterized by distractibility, disorganization, and difficulty sustaining focus [94]. Attention impairment has been previously linked to depression and suicidal history, and may also increase heat vulnerability by limiting a child’s ability to recognize and respond appropriately to heat-related discomfort [95]. Children with attentional deficits may be less likely to engage in adaptive behaviors such as staying hydrated, seeking shade, or taking cooling breaks, and may have greater difficulty adhering to caregiver guidance during extreme heat [88,96]. Attention impairment in children with ADHD can contribute to mental exhaustion and executive dysfunction [95]. Managing daily tasks, social interactions, activities, and school work can feel overwhelming, and difficulty in managing this “load” may make children more vulnerable to additional stressors like heat, which further taxes attentional resources. Coping strategies may simultaneously decline, and over time, this persistent strain, frustration, and inability to adapt could foster negative self-perceptions and rumination, processes strongly linked to the development of depression [97]. These behavioral and cognitive challenges can exacerbate physiological stress and compound mental health risks, suggesting that mechanisms beyond impulsivity contribute to the observed associations.

While our results support extending the Differential Susceptibility Model to physical environmental stressors like extreme heat, the findings suggest a more nuanced relationship than simple differential susceptibility [43]. Children with the inattentive subtype showed nearly three times higher risk for overlapping MDD-suicidal presentations during heatwaves, we also found elevated risks for those with unspecified ADHD subtypes across multiple outcomes. Furthermore, the sex differences observed, with females showing greater vulnerability for suicide outcomes and males for MDD, suggest that numerous interacting factors beyond inherent plasticity markers are at play. These results align more closely with an intersectional vulnerability model where multiple risk factors (biological, clinical, and social identities) combine to create unique risk profiles. Rather than supporting a single theoretical framework, our findings highlight the need for integrated models that consider neurobiological sensitivity and intersecting social identities.

Although our findings suggest increased risk during heatwaves, we also observed that in some cases cumulative exposure appeared to have a protective effect, particularly among White males and those with inattentive ADHD. These findings may indicate real behavioral or contextual differences. For instance, cumulative heat exposure could alter daily activities (e.g., reduced physical activity, increased caregiver supervision, or caregiver-imposed breaks from the heat or additional hydration measures) that mitigate mental health consequences in certain groups. Future research is needed to test whether these apparent protective effects represent contextual adaptation or statistical artifact.

### Strengths and limitations

A key strength of this study is the use of heatwave exposure as the primary variable, aligning with public health frameworks that classify heatwaves as prolonged hazardous events rather than isolated temperature spikes. This approach is particularly relevant for community-level preparedness and intervention efforts. We applied a standardized definition of a heatwave (three consecutive days of elevated temperatures) that accounts for both local county-level climatology and acclimatization using EHF. Future research should refine exposure metrics by considering heatwave duration and severity, as well as absolute temperature thresholds, when morbidity may spike. Another methodological strength is the matched case design. This design effectively accounts for transient environmental exposures and short-term health outcomes by matching cases to reference periods, thereby controlling for confounding by county-level and seasonal factors.

Despite these strengths, the study has important limitations. While we acknowledge that administrative data are not a clinical gold standard, recent validation research supports their utility for identifying neurodevelopmental disorders, including ADHD, with reasonably high accuracy [98]. Our study highlights the advantages of utilizing large-scale administrative data for population-based research, including cost-effectiveness, longitudinal tracking, and feasibility for studies of outcomes like ADHD, while also acknowledging inherent limitations such as potential misclassification and the lack of rich clinical detail [98]. Our administrative ED data do not contain longitudinal prior records linked to the index visit, nor do they include ADHD severity, symptom scales, or treatment data (e.g., medication use), limiting our ability to examine clinical course and treatment-related effect modification. Additionally, although using any diagnosis position increases capture of comorbid ADHD in ED encounters, it may also include historical diagnoses; this is an inherent tradeoff in administrative datasets.

Given the small case counts in certain subgroups, intersectional estimates should be considered hypothesis-generating and interpreted with caution until replicated in larger, independent datasets. ADHD subtype classifications in this study relied on ICD-9 coding in electronic health records, which do not distinguish between combined and unspecified presentations, and are often carried over from prior problem lists rather than reassessed during each clinical encounter. This inconsistency may introduce misclassification bias. And it is unclear whether subtype distinctions meaningfully contribute to our findings beyond the broader ADHD diagnosis. We fully recognize concerns about the reliability and stability of these classifications; however, our goal in presenting results by ADHD presentation was not to assert the subtypes as fixed diagnostic entities, but to explore potential heterogeneity in environmental sensitivity among youth with differing symptom profiles. While imperfect, these clinician-assigned subtypes may reflect real-world differences in symptom severity or concern during crisis presentation that could plausibly relate to heat vulnerability. Future studies should consider whether focusing on ADHD as a whole, rather than specific subtypes, provides a more reliable analytic approach.

Similarly, the identification of major depressive disorder (MDD) in EHR data presents challenges. A formal MDD diagnosis requires symptoms to persist for at least two weeks, yet emergency department (ED) encounters often lack sufficient longitudinal information to confirm this criterion. Without access to outpatient or longitudinal mental health records, it is difficult to determine whether cases classified as MDD in our dataset fully meet diagnostic thresholds. Cases of MDD that are diagnosed in the ED are also likely the most severe cases and therefore underestimates the true prevalence of MDD in the community. While estimates for younger children should be interpreted with caution due to small sample size, they still provide valuable insight into early-onset vulnerability. These limitations underscore the need for additional data sources, such as primary care or outpatient records, to enhance diagnostic validity. Additionally, parasuicidal behavior (deliberate self-harm behavior absent the intent to die), while clinically meaningful, is challenging to capture using ICD-9 codes and was not addressed in the studies that informed our outcome selection. Future research should investigate parasuicidal behavior in relation to environmental stressors, like heat waves. Lastly, the lack of information on medication status limits insights into the potential influence of stimulants or other psychotropic medications on heat sensitivity.

## Conclusion

Our findings highlight the complex interplay of environmental and individual risk factors in shaping heat vulnerability in children with ADHD. By identifying high-risk subgroups, this study highlights the need for targeted interventions to mitigate the mental health impacts of extreme heat. Future research should investigate underlying mechanisms and long-term effects, including other clinically relevant outcomes such as accidental injuries and risk-taking behaviors that may be exacerbated by heat exposure. Public health efforts should prioritize adaptive strategies to protect at-risk pediatric populations from climate-related stressors. The southeastern U.S. is projected to experience more frequent and intense extreme heat events, potentially increasing the mental health burden on vulnerable pediatric populations. Schools should implement heat action plans that include mental and behavioral health monitoring during extreme heat events, with particular attention on students with ADHD.

## Supporting information

S1 Fig Forest plot of Poisson mixed-effect models estimating risk of an ED visit for MDD varied across intersecting identities of race, gender, and ADHD subtype. (DOCX)

S2 Fig Forest plot of Poisson mixed-effect models estimating risk of an ED visit for suicidal behavior varied across intersecting identities of race, gender, and ADHD subtype. (DOCX)

S3 Fig Forest plot of Poisson mixed-effect models estimating risk of an ED visit for overlapping MDD and suicidal behavior varied across intersecting identities of race, gender, and ADHD subtype. (DOCX)

S1 Table ICD 9 and ICD 10 codes used to define ADHD, ADHD subtypes, MDD, and suicidal behavior. ADHD combined and other, unspecified subtypes were not defined during the use of ICD-9 codes (2008–2015).
(DOCX)

S2 Table Average statistics of heatwave days for all counties in North Carolina between 2008 and 2021. (DOCX)

S3 Table Relative risk (RR), 95% confidence intervals (95% CI), and p-value (p) from the sensitivity analysis, where the analysis was limited to cases occurring in the warm season only. Poisson mixed-effect regression models for same-day, lagged, and cumulative effects were analyzed for each outcome. Models were also run to assess effect modification across covariates.
(DOCX)

S4 Table Relative risk (RR), 95% confidence intervals (95% CI), and p-value (p) from the sensitivity analysis, examining ADHD-related ED visits defined by either a primary or secondary diagnosis of ADHD. Poisson mixed-effect regression models were run for same-day, lagged, and cumulative effects. Models were also run to assess effect modification across covariates.
(DOCX)

## References

[1] Thapar A, Cooper M. Attention deficit hyperactivity disorder. Lancet. 2016;387(10024):1240–50. 26386541 10.1016/S0140-6736(15)00238-X

[2] Verkuijl N, Perkins M, Fazel M. Childhood attention-deficit/hyperactivity disorder. BMJ. 2015;350:h2168. 10.1136/bmj.h2168 25994532

[3] Xu G, Strathearn L, Liu B, Yang B, Bao W. Twenty-Year Trends in Diagnosed Attention-Deficit/Hyperactivity Disorder Among US Children and Adolescents, 1997-2016. JAMA Netw Open. 2018;1(4):e181471. doi: 10.1001/jamanetworkopen.2018.1471 PMC6324288 30646132

[4] Hinshaw SP, Nguyen PT, O’Grady SM, Rosenthal EA. Annual Research Review: Attention-deficit/hyperactivity disorder in girls and women: underrepresentation, longitudinal processes, and key directions. J Child Psychol Psychiatry. 2022;63(4):484–96. doi: 10.1111/jcpp.13480 34231220

[5] Young S, Adamo N, Ásgeirsdóttir BB, Branney P, Beckett M, Colley W, et al. Females with ADHD: An expert consensus statement taking a lifespan approach providing guidance for the identification and treatment of attention-deficit/ hyperactivity disorder in girls and women. BMC Psychiatry. 2020;20(1):404. doi: 10.1186/s12888-020-02707-9 32787804 PMC7422602

[6] Danielson ML, Bitsko RH, Ghandour RM, Holbrook JR, Kogan MD, Blumberg SJ. Prevalence of Parent-Reported ADHD Diagnosis and Associated Treatment Among U.S. Children and Adolescents, 2016. J Clin Child Adolesc Psychol. 2018;47(2):199–212. doi: 10.1080/15374416.2017.1417860 29363986 PMC5834391

[7] CDC. Data and Statistics on ADHD. Attention-Deficit/Hyperactivity Disorder (ADHD). 2024. https://www.cdc.gov/adhd/data/index.html

[8] Stewman CG, Liebman C, Fink L, Sandella B. Attention Deficit Hyperactivity Disorder: Unique Considerations in Athletes. Sports Health. 2018;10(1):40–6. doi: 10.1177/1941738117742906 29144831 PMC5753970

[9] Rowland AS, Skipper B, Rabiner DL, Umbach DM, Stallone L, Campbell RA, et al. The shifting subtypes of ADHD: classification depends on how symptom reports are combined. J Abnorm Child Psychol. 2008;36(5):731–43. doi: 10.1007/s10802-007-9203-7 18347973 PMC3646478

[10] Klein-Schwartz W. Abuse and toxicity of methylphenidate. Curr Opin Pediatr. 2002;14(2):219–23. doi: 10.1097/00008480-200204000-00013 11981294

[11] May DE, Kratochvil CJ. Attention-deficit hyperactivity disorder: recent advances in paediatric pharmacotherapy. Drugs. 2010;70(1):15–40. doi: 10.2165/11530540-000000000-00000 20030423

[12] Swart J, Lamberts RP, Lambert MI, St Clair Gibson A, Lambert EV, Skowno J, et al. Exercising with reserve: evidence that the central nervous system regulates prolonged exercise performance. Br J Sports Med. 2009;43(10):782–8. doi: 10.1136/bjsm.2008.055889 19052141

[13] Allen SB, Cross KP. Out of the frying pan, into the fire: a case of heat shock and its fatal complications. Pediatr Emerg Care. 2014;30(12):904–10. doi: 10.1097/PEC.0000000000000296 25469604

[14] Hinshaw SP, Owens EB, Zalecki C, Huggins SP, Montenegro-Nevado AJ, Schrodek E, et al. Prospective follow-up of girls with attention-deficit/hyperactivity disorder into early adulthood: continuing impairment includes elevated risk for suicide attempts and self-injury. J Consult Clin Psychol. 2012;80(6):1041–51. doi: 10.1037/a0029451 22889337 PMC3543865

[15] Carter LM, Terando A, Dow K, Hiers K, Kunkel KE, Lascurain A, et al. Southeast. Impacts, Risks, and Adaptation in the United States: The Fourth National Climate Assessment, Volume II. U.S. Global Change Research Program. 2018.

[16] Diem JE, Stauber CE, Rothenberg R. Heat in the southeastern United States: Characteristics, trends, and potential health impact. PLoS One. 2017;12(5):e0177937. doi: 10.1371/journal.pone.0177937 28520817 PMC5433771

[17] Barbaresi WJ, Colligan RC, Weaver AL, Voigt RG, Killian JM, Katusic SK. Mortality, ADHD, and psychosocial adversity in adults with childhood ADHD: a prospective study. Pediatrics. 2013;131(4):637–44. doi: 10.1542/peds.2012-2354 23460687 PMC3821174

[18] Chronis-Tuscano A, Molina BSG, Pelham WE, Applegate B, Dahlke A, Overmyer M, et al. Very early predictors of adolescent depression and suicide attempts in children with attention-deficit/hyperactivity disorder. Arch Gen Psychiatry. 2010;67(10):1044–51. doi: 10.1001/archgenpsychiatry.2010.127 20921120 PMC3382065

[19] Furczyk K, Thome J. Adult ADHD and suicide. Atten Defic Hyperact Disord. 2014;6(3):153–8. doi: 10.1007/s12402-014-0150-1 25063344

[20] Goldston DB, Daniel SS, Erkanli A, Reboussin BA, Mayfield A, Frazier PH, et al. Psychiatric diagnoses as contemporaneous risk factors for suicide attempts among adolescents and young adults: developmental changes. J Consult Clin Psychol. 2009;77(2):281–90. doi: 10.1037/a0014732 19309187 PMC2819300

[21] Hurtig T, Taanila A, Moilanen I, Nordström T, Ebeling H. Suicidal and self-harm behaviour associated with adolescent attention deficit hyperactivity disorder-a study in the Northern Finland Birth Cohort 1986. Nord J Psychiatry. 2012;66(5):320–8. doi: 10.3109/08039488.2011.644806 22242914

[22] Murphy KR, Barkley RA, Bush T. Young adults with attention deficit hyperactivity disorder: subtype differences in comorbidity, educational, and clinical history. J Nerv Ment Dis. 2002;190(3):147–57. doi: 10.1097/00005053-200203000-00003 11923649

[23] Rohn RD, Sarles RM, Kenny TJ, Reynolds BJ, Heald FP. Adolescents who attempt suicide. J Pediatr. 1977;90(4):636–8. doi: 10.1016/s0022-3476(77)80389-2 839384

[24] Manor I, Gutnik I, Ben-Dor DH, Apter A, Sever J, Tyano S, et al. Possible association between attention deficit hyperactivity disorder and attempted suicide in adolescents - a pilot study. Eur Psychiatry. 2010;25(3):146–50. doi: 10.1016/j.eurpsy.2009.06.001 19699060

[25] Garas P, Balazs J. Long-Term Suicide Risk of Children and Adolescents With Attention Deficit and Hyperactivity Disorder-A Systematic Review. Front Psychiatry. 2020;11:557909. doi: 10.3389/fpsyt.2020.557909 33408650 PMC7779592

[26] Nasser EH, Overholser JC. Assessing varying degrees of lethality in depressed adolescent suicide attempters. Acta Psychiatr Scand. 1999;99(6):423–31. doi: 10.1111/j.1600-0447.1999.tb00988.x 10408264

[27] Dekkers TJ, Popma A, Agelink van Rentergem JA, Bexkens A, Huizenga HM. Risky decision making in Attention-Deficit/Hyperactivity Disorder: A meta-regression analysis. Clin Psychol Rev. 2016;45:1–16. doi: 10.1016/j.cpr.2016.03.001 26978323

[28] Dekkers TJ, Hornstra R, van der Oord S, Luman M, Hoekstra PJ, Groenman AP, et al. Meta-analysis: Which Components of Parent Training Work for Children With Attention-Deficit/Hyperactivity Disorder?. J Am Acad Child Adolesc Psychiatry. 2022;61(4):478–94. doi: 10.1016/j.jaac.2021.06.015 34224837

[29] Miller AC, Keenan JM, Betjemann RS, Willcutt EG, Pennington BF, Olson RK. Reading comprehension in children with ADHD: cognitive underpinnings of the centrality deficit. J Abnorm Child Psychol. 2013;41(3):473–83. doi: 10.1007/s10802-012-9686-8 23054132 PMC3561476

[30] Pollak Y, Dekkers TJ, Shoham R, Huizenga HM. Risk-Taking Behavior in Attention Deficit/Hyperactivity Disorder (ADHD): a Review of Potential Underlying Mechanisms and of Interventions. Curr Psychiatry Rep. 2019;21(5):33. doi: 10.1007/s11920-019-1019-y 30903380

[31] Boyer TW. The development of risk-taking: A multi-perspective review. Dev Rev. 2006;26(3):291–345.

[32] Hsiang SM, Burke M, Miguel E. Quantifying the influence of climate on human conflict. Science. 2013;341(6151):1235367. doi: 10.1126/science.1235367 24031020

[33] Bitsko RH, Holbrook JR, Robinson LR, Kaminski JW, Ghandour R, Smith C. Health care, family, and community factors associated with mental, behavioral, and developmental disorders in early childhood — United States, 2011–2012. MMWR Morb Mortal Wkly Rep. 2016;65(9):221–6. 26963052 10.15585/mmwr.mm6509a1

[34] Sayal K, Prasad V, Daley D, Ford T, Coghill D. ADHD in children and young people: prevalence, care pathways, and service provision. Lancet Psychiatry. 2018;5(2):175–86. doi: 10.1016/S2215-0366(17)30167-0 29033005

[35] Harlan SL, Declet-Barreto JH, Stefanov WL, Petitti DB. Neighborhood effects on heat deaths: social and environmental predictors of vulnerability in Maricopa County, Arizona. Environ Health Perspect. 2013;121(2):197–204. doi: 10.1289/ehp.1104625 23164621 PMC3569676

[36] Anderson GB, Bell ML. Heat waves in the United States: mortality risk during heat waves and effect modification by heat wave characteristics in 43 U.S. communities. Environ Health Perspect. 2011;119(2):210–8. doi: 10.1289/ehp.1002313 21084239 PMC3040608

[37] Casey JA, James P, Cushing L, Jesdale BM, Morello-Frosch R. Race, Ethnicity, Income Concentration and 10-Year Change in Urban Greenness in the United States. Int J Environ Res Public Health. 2017;14(12):1546. doi: 10.3390/ijerph14121546 29232867 PMC5750964

[38] Hinshaw SP. The stigmatization of mental illness in children and parents: developmental issues, family concerns, and research needs. J Child Psychol Psychiatry. 2005;46(7):714–34. doi: 10.1111/j.1469-7610.2005.01456.x 15972067

[39] Alegria M, Vallas M, Pumariega AJ. Racial and ethnic disparities in pediatric mental health. Child Adolesc Psychiatr Clin N Am. 2010;19(4):759–74. doi: 10.1016/j.chc.2010.07.001 21056345 PMC3011932

[40] Martin J. Why are females less likely to be diagnosed with ADHD in childhood than males?. Lancet Psychiatry. 2024;11(4):303–10. doi: 10.1016/S2215-0366(24)00010-5 38340761

[41] Skogli EW, Teicher MH, Andersen PN, Hovik KT, Øie M. ADHD in girls and boys--gender differences in co-existing symptoms and executive function measures. BMC Psychiatry. 2013;13:298. doi: 10.1186/1471-244X-13-298 24206839 PMC3827008

[42] Clark A, Grineski S, Curtis DS, Cheung ESL. Identifying groups at-risk to extreme heat: Intersections of age, race/ethnicity, and socioeconomic status. Environ Int. 2024;191:108988. doi: 10.1016/j.envint.2024.108988 39217722 PMC11569890

[43] Belsky J, Pluess M. Beyond diathesis stress: differential susceptibility to environmental influences. Psychol Bull. 2009;135(6):885–908. doi: 10.1037/a0017376 19883141

[44] Smeekens S, Riksen-Walraven JM, van Bakel HJA. Multiple determinants of externalizing behavior in 5-year-olds: a longitudinal model. J Abnorm Child Psychol. 2007;35(3):347–61. doi: 10.1007/s10802-006-9095-y 17243016 PMC1915644

[45] Belsky J. Differential Susceptibility to Environmental Influences. ICEP. 2013;7(2):15–31. doi: 10.1007/2288-6729-7-2-15

[46] Lengua LJ, Wolchik SA, Sandler IN, West SG. The additive and interactive effects of parenting and temperament in predicting adjustment problems of children of divorce. J Clin Child Psychol. 2000;29(2):232–44. doi: 10.1207/S15374424jccp2902_9 10802832

[47] NC Hospital Discharge Data. NC Hospital Discharge Data. https://www.shepscenter.unc.edu/data/nc-hospital-discharge-data/

[48] Patros CHG, Hudec KL, Alderson RM, Kasper LJ, Davidson C, Wingate LR. Symptoms of attention-deficit/hyperactivity disorder (ADHD) moderate suicidal behaviors in college students with depressed mood. J Clin Psychol. 2013;69(9):980–93. doi: 10.1002/jclp.21994 23775306

[49] Nori-Sarma A, Sun S, Sun Y, Spangler KR, Oblath R, Galea S. Association Between Ambient Heat and Risk of Emergency Department Visits for Mental Health Among US Adults, 2010 to 2019. JAMA Psychiatry. 2022;79(4):341–9. 35195664 10.1001/jamapsychiatry.2021.4369 PMC8867392

[50] Runkle JD, Sugg MM, Berry A, Reed C, Cowan K, Wertis L, et al. Association of Psychiatric Emergency Visits and Warm Ambient Temperature during Pregnancy: A Time-Stratified Case-Crossover Study. Environ Health Perspect. 2024;132(6):67001. doi: 10.1289/EHP13293 38829735 PMC11166382

[51] First MB, Rebello TJ, Keeley JW, Bhargava R, Dai Y, Kulygina M, et al. Do mental health professionals use diagnostic classifications the way we think they do? A global survey. World Psychiatry. 2018;17(2):187–95. doi: 10.1002/wps.20525 29856559 PMC5980454

[52] Kerr AJ, Wang TKM, Jiang Y, Grey C, Wells S, Poppe KK. The importance of considering both primary and secondary diagnostic codes when using administrative health data to study acute coronary syndrome epidemiology (ANZACS-QI 47). Eur Heart J - Qual Care Clin Outcomes. 2021;7(6):548–55. 32592466 10.1093/ehjqcco/qcaa056

[53] Chung W, Jiang S-F, Paksarian D, Nikolaidis A, Castellanos FX, Merikangas KR, et al. Trends in the Prevalence and Incidence of Attention-Deficit/Hyperactivity Disorder Among Adults and Children of Different Racial and Ethnic Groups. JAMA Netw Open. 2019;2(11):e1914344. doi: 10.1001/jamanetworkopen.2019.14344 31675080 PMC6826640

[54] Adkins-Jackson PB, Chantarat T, Bailey ZD, Ponce NA. Measuring structural racism: a guide for epidemiologists and other health researchers. Am J Epidemiol. 2022;191(4). 10.1093/aje/kwab239 PMC9077112 34564723

[55] Ryder S. A bridge to challenging environmental inequality: intersectionality, environmental justice, and disaster vulnerability. https://kuscholarworks.ku.edu/entities/publication/1e6efa2d-3f07-42f0-824f-eeef8483816e

[56] Alvarez CH, Evans CR. Intersectional environmental justice and population health inequalities: A novel approach. Soc Sci Med. 2021;269:113559. doi: 10.1016/j.socscimed.2020.113559 33309156

[57] Rapoport IL, Groenman AP. A Review of Sex and Gender Factors in Stimulant Treatment for ADHD: Knowledge Gaps and Future Directions. J Atten Disord. 2025;29(8):602–16. doi: 10.1177/10870547251315601 39878255 PMC12064863

[58] Hansen A, Bi P, Nitschke M, Ryan P, Pisaniello D, Tucker G. The effect of heat waves on mental health in a temperate Australian city. Environ Health Perspect. 2008;116(10):1369–75. doi: 10.1289/ehp.11339 18941580 PMC2569097

[59] Anderson BG, Bell ML. Weather-related mortality: how heat, cold, and heat waves affect mortality in the United States. Epidemiology. 2009;20(2):205–13. doi: 10.1097/EDE.0b013e318190ee08 19194300 PMC3366558

[60] Rocklov J, Barnett AG, Woodward A. On the estimation of heat-intensity and heat-duration effects in time series models of temperature-related mortality in Stockholm, Sweden. Environ Health. 2012;11:23. doi: 10.1186/1476-069X-11-23 22490779 PMC3431980

[61] Rony MKK, Alamgir HM. High temperatures on mental health: Recognizing the association and the need for proactive strategies-A perspective. Health Sci Rep. 2023;6(12):e1729. doi: 10.1002/hsr2.1729 38059052 PMC10696165

[62] Durre I, Arguez A, Schreck CJ, Squires MF, Vose RS. Daily High-Resolution Temperature and Precipitation Fields for the Contiguous United States from 1951 to Present. Journal of Atmospheric and Oceanic Technology. 2022;39(12):1837–55. doi: 10.1175/jtech-d-22-0024.1

[63] Nairn JR, Fawcett RJB. The excess heat factor: a metric for heatwave intensity and its use in classifying heatwave severity. Int J Environ Res Public Health. 2014;12(1):227–53. doi: 10.3390/ijerph120100227 25546282 PMC4306859

[64] Scalley BD, Spicer T, Jian L, Xiao J, Nairn J, Robertson A, et al. Responding to heatwave intensity: Excess Heat Factor is a superior predictor of health service utilisation and a trigger for heatwave plans. Aust N Z J Public Health. 2015;39(6):582–7. doi: 10.1111/1753-6405.12421 26260877

[65] Bobb JF, Obermeyer Z, Wang Y, Dominici F. Cause-specific risk of hospital admission related to extreme heat in older adults. JAMA. 2014;312(24):2659–67. doi: 10.1001/jama.2014.15715 25536257 PMC4319792

[66] Burrows K, Anderson GB, Yan M, Wilson A, Sabath MB, Son JY, et al. Health disparities among older adults following tropical cyclone exposure in Florida. Nat Commun. 2023;14(1):2221. doi: 10.1038/s41467-023-37675-7 37076480 PMC10115860

[67] Liu JC, Wilson A, Mickley LJ, Dominici F, Ebisu K, Wang Y, et al. Wildfire-specific Fine Particulate Matter and Risk of Hospital Admissions in Urban and Rural Counties. Epidemiology. 2017;28(1):77–85. doi: 10.1097/EDE.0000000000000556 27648592 PMC5130603

[68] Parks RM, Benavides J, Anderson GB, Nethery RC, Navas-Acien A, Dominici F. Association of Tropical Cyclones With County-Level Mortality in the US. JAMA. 2022;327(10):946–55. 35258534 10.1001/jama.2022.1682 PMC8905400

[69] Cheng B-J, Li H, Meng K, Li T-L, Meng X-C, Wang J, et al. Short-term effects of heatwaves on clinical and subclinical cardiovascular indicators in Chinese adults: A distributed lag analysis. Environ Int. 2024;183:108358. doi: 10.1016/j.envint.2023.108358 38056095

[70] Dang TN, Honda Y, Van Do D, Pham ALT, Chu C, Huang C. Effects of extreme temperatures on mortality and hospitalization in Ho Chi Minh City, Vietnam. Int J Environ Res Public Health. 2019;16(3):432. 30717328 10.3390/ijerph16030432 PMC6388260

[71] Hu J, Wen Y, Duan Y, Yan S, Liao Y, Pan H, et al. The impact of extreme heat and heat waves on emergency ambulance dispatches due to external cause in Shenzhen, China. Environ Pollut. 2020;261:114156. doi: 10.1016/j.envpol.2020.114156 32092626

[72] Ulrich SE, Sugg MM, Guignet D, Runkle JD. Mental health disparities among maternal populations following heatwave exposure in North Carolina (2011–2019): a matched analysis. Lancet Reg Health - Am. 2025;42:100998. 39925466 10.1016/j.lana.2025.100998 PMC11804822

[73] SAS Institute Inc. SAS/STAT 9.3 User’s Guide: The GLIMMIX Procedure. SAS Institute. 2011.

[74] Breslow NE, Clayton DG. Approximate Inference in Generalized Linear Mixed Models. J Am Stat Assoc. 1993;88(421):9–25.

[75] Benjamini Y, Hochberg Y. Controlling the False Discovery Rate: A Practical and Powerful Approach to Multiple Testing. J R Stat Soc Ser B Stat Methodol. 1995;57(1):289–300.

[76] SAS Institute Inc. SAS Version 9.4. Cary, NC: SAS Inst Inc. 2024.

[77] Deng X, Brotzge J, Tracy M, Chang HH, Romeiko X, Zhang W, et al. Identifying joint impacts of sun radiation, temperature, humidity, and rain duration on triggering mental disorders using a high-resolution weather monitoring system. Environ Int. 2022;167:107411. doi: 10.1016/j.envint.2022.107411 35870379 PMC12184810

[78] Qiu X, Danesh-Yazdi M, Wei Y, Di Q, Just A, Zanobetti A, et al. Associations of short-term exposure to air pollution and increased ambient temperature with psychiatric hospital admissions in older adults in the USA: a case-crossover study. Lancet Planet Health. 2022;6(4):e331–41. doi: 10.1016/S2542-5196(22)00017-1 35397221 PMC9044858

[79] Wang X, Lavigne E, Ouellette-kuntz H, Chen BE. Acute impacts of extreme temperature exposure on emergency room admissions related to mental and behavior disorders in Toronto, Canada. J Affect Disord. 2014;155:154–61. doi: 10.1016/j.jad.2013.10.042 24332428

[80] Liu J, Varghese BM, Hansen A, Xiang J, Zhang Y, Dear K, et al. Is there an association between hot weather and poor mental health outcomes? A systematic review and meta-analysis. Environ Int. 2021;153:106533. doi: 10.1016/j.envint.2021.106533 33799230

[81] Bernstein AS, Sun S, Weinberger KR, Spangler KR, Sheffield PE, Wellenius GA. Warm Season and Emergency Department Visits to U.S. Children’s Hospitals. Environ Health Perspect. 2022;130(1):17001. doi: 10.1289/EHP8083 35044241 PMC8767980

[82] Corcuera Hotz I, Hajat S. The Effects of Temperature on Accident and Emergency Department Attendances in London: A Time-Series Regression Analysis. Int J Environ Res Public Health. 2020;17(6):1957. doi: 10.3390/ijerph17061957 32192045 PMC7142952

[83] Girma B, Liu B, Schinasi LH, Clougherty JE, Sheffield PE. High ambient temperatures associations with children and young adult injury emergency department visits in NYC. Environ Res Health. 2023;1(3):035004. doi: 10.1088/2752-5309/ace27b 37448837 PMC10336474

[84] Stowell JD, Sun Y, Spangler KR, Milando CW, Bernstein A, Weinberger KR, et al. Warm-season temperatures and emergency department visits among children with health insurance. Environ Res Health. 2023;1(1):015002. doi: 10.1088/2752-5309/ac78fa 36337257 PMC9623446

[85] Shoib S, Hussaini SS, Armiya’u AY, Saeed F, Őri D, Roza TH, et al. Prevention of suicides associated with global warming: perspectives from early career psychiatrists. Front Psychiatry. 2023;14:1251630. doi: 10.3389/fpsyt.2023.1251630 38045615 PMC10693336

[86] Cheshire WP, Fealey RD. Drug-induced hyperhidrosis and hypohidrosis: incidence, prevention and management. Drug Saf. 2008;31(2):109–26. doi: 10.2165/00002018-200831020-00002 18217788

[87] Ljung T, Chen Q, Lichtenstein P, Larsson H. Common etiological factors of attention-deficit/hyperactivity disorder and suicidal behavior: a population-based study in Sweden. JAMA Psychiatry. 2014;71(8):958–64. doi: 10.1001/jamapsychiatry.2014.363 24964928

[88] Nigg JT. Attention-deficit/hyperactivity disorder and adverse health outcomes. Clin Psychol Rev. 2013;33(2):215–28. doi: 10.1016/j.cpr.2012.11.005 23298633 PMC4322430

[89] Balazs J, Kereszteny A. Attention-deficit/hyperactivity disorder and suicide: A systematic review. World J Psychiatry. 2017;7(1):44–59. doi: 10.5498/wjp.v7.i1.44 28401048 PMC5371172

[90] Price JH, Foh EP. Descriptive Epidemiology of Female Suicides by Race and Ethnicity. J Community Health. 2024;49(6):1054–61. doi: 10.1007/s10900-024-01368-z 38853209 PMC11413112

[91] Carter SP, Campbell SB, Wee JY, Law KC, Lehavot K, Simpson T, et al. Suicide Attempts Among Racial and Ethnic Groups in a Nationally Representative Sample. J Racial Ethn Health Disparities. 2022;9(5):1783–93. doi: 10.1007/s40615-021-01115-3 34291440 PMC8294284

[92] Austgulen A, Skram NKG, Haavik J, Lundervold AJ. Risk factors of suicidal spectrum behaviors in adults and adolescents with attention-deficit / hyperactivity disorder - a systematic review. BMC Psychiatry. 2023;23(1):612. doi: 10.1186/s12888-023-05099-8 37605105 PMC10441735

[93] Beauchaine TP, Hinshaw SP, Bridge JA. Nonsuicidal Self-Injury and Suicidal Behaviors in Girls: The Case for Targeted Prevention in Preadolescence. Clin Psychol Sci. 2019;7(4):643–67. doi: 10.1177/2167702618818474 31485384 PMC6726409

[94] Substance Abuse and Mental Health Services Administration. DSM-5 Changes: Implications for Child Serious Emotional Disturbance. Rockville (MD): Substance Abuse and Mental Health Services Administration (US). 2016. http://www.ncbi.nlm.nih.gov/books/NBK519708/30199184

[95] Keilp JG, Gorlyn M, Russell M, Oquendo MA, Burke AK, Harkavy-Friedman J, et al. Neuropsychological function and suicidal behavior: attention control, memory and executive dysfunction in suicide attempt. Psychol Med. 2013;43(3):539–51. doi: 10.1017/S0033291712001419 22781400 PMC3767483

[96] Barkley RA. Recent longitudinal studies of childhood attention-deficit/hyperactivity disorder: Important themes and questions for further research. J Abnorm Psychol. 2016;125(2):248–55. doi: 10.1037/abn0000125 26854509

[97] Kovacs M, Goldston D. Cognitive and social cognitive development of depressed children and adolescents. J Am Acad Child Adolesc Psychiatry. 1991;30(3):388–92. doi: 10.1097/00004583-199105000-00006 1711524

[98] Straub L, Bateman BT, Hernandez-Diaz S, York C, Zhu Y, Suarez EA, et al. Validity of claims-based algorithms to identify neurodevelopmental disorders in children. Pharmacoepidemiol Drug Saf. 2021;30(12):1635–42. doi: 10.1002/pds.5369 34623720 PMC8578450

