# Supplementary material for: Assessing the impact of heatwaves on emergency visits for major depression and suicidal ideation in youth with attention-deficit/hyperactivity disorder
Source: PLOS Ment Health. 2025 Oct 29;2(10):e0000444. doi: 10.1371/journal.pmen.0000444 (PMC12798237; doi:10.1371/journal.pmen.0000444)
Supplement: S3 Fig — (DOCX) [file pmen.0000444.s003.docx]

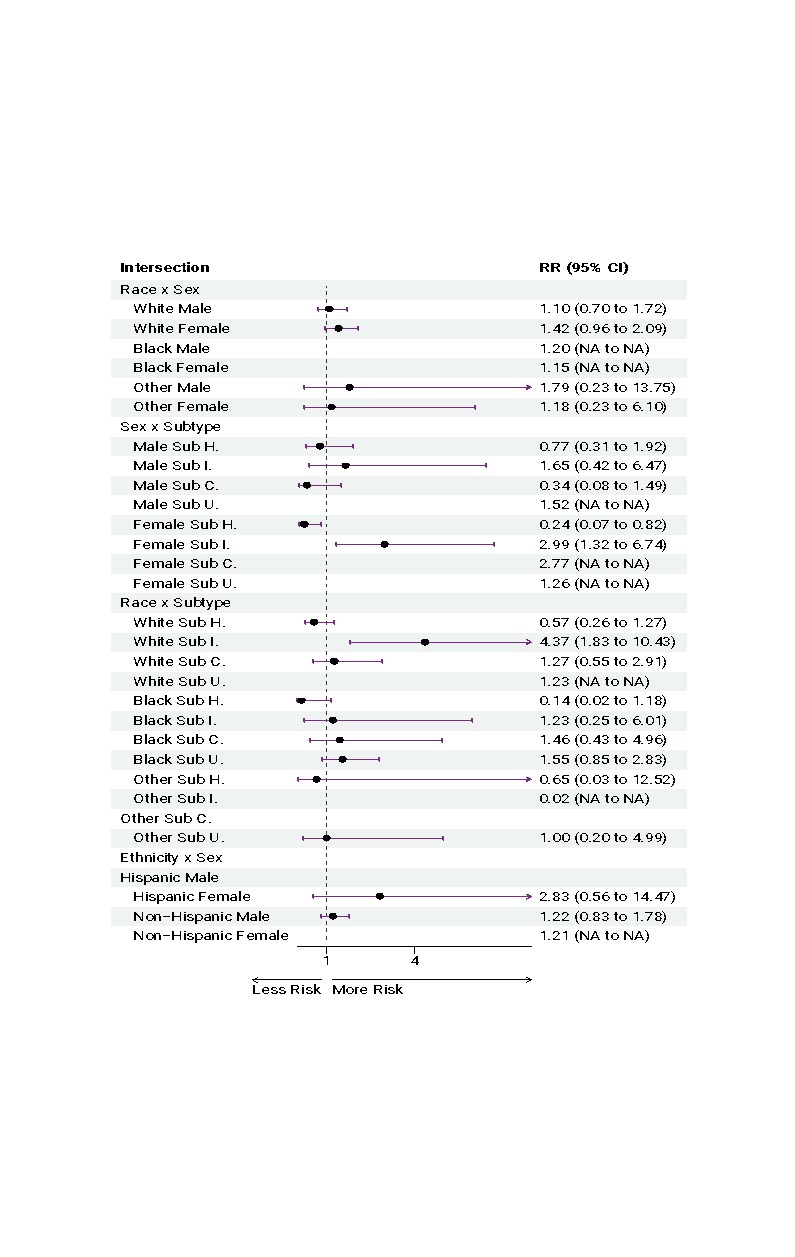


S3 Fig. Forest plot of Poisson mixed-effect models estimating risk of an ED visit for overlapping MDD and suicidal behavior varied across intersecting identities of race, gender, and ADHD subtype
