# Supplementary material for: Assessing the impact of heatwaves on emergency visits for major depression and suicidal ideation in youth with attention-deficit/hyperactivity disorder
Source: PLOS Ment Health. 2025 Oct 29;2(10):e0000444. doi: 10.1371/journal.pmen.0000444 (PMC12798237; doi:10.1371/journal.pmen.0000444)
Supplement: S1 Table — ADHD combined and other, unspecified subtypes were not defined during the use of ICD-9 codes (2008–2015). (DOCX) [file pmen.0000444.s004.docx]

|  | ICD-9 | ICD-10 |
| --- | --- | --- |
| Attention deficit hyperactivity disorder (ADHD) | 314 | F90 |
| Major depressive disorder (MDD) | 296.3 | F33 |
| Suicidal behavior (SUIC) | E95, V628.4 | X60-X84, R45.851, T14.91 |
| ADHD subtype: hyperactive | 314.01 | F90.1 |
| ADHD subtype: inattentive | 314.00 | F90.0 |
| ADHD subtype: combined | NA | F90.2 |
| ADHD subtype: other, unspecified | NA | F90.8, F90.9 |

S1 Table . ICD 9 and ICD 10 codes used to define ADHD, ADHD subtypes, MDD, and suicidal behavior. ADHD combined and other, unspecified subtypes were not defined during the use of ICD-9 codes (2008-2015).
