# Supplementary material for: Assessing the impact of heatwaves on emergency visits for major depression and suicidal ideation in youth with attention-deficit/hyperactivity disorder
Source: PLOS Ment Health. 2025 Oct 29;2(10):e0000444. doi: 10.1371/journal.pmen.0000444 (PMC12798237; doi:10.1371/journal.pmen.0000444)
Supplement: S2 Table — (DOCX) [file pmen.0000444.s005.docx]

| **County** | **Heatwave Days** | | | |
| --- | --- | --- | --- | --- |
|  | Mean (SD) | Median (IQR) | Min | Max |
| Alamance | 27.7 (1) | 27.6 (1.3) | 17.1 | 38.7 |
| Alexander | 26.5 (0.9) | 26.5 (1.1) | 15.4 | 37.9 |
| Alleghany | 22.9 (0.9) | 22.9 (1.1) | 11.4 | 34.8 |
| Anson | 28.6 (0.9) | 28.6 (1.2) | 18.3 | 40.8 |
| Ashe | 22.1 (0.9) | 22 (1.2) | 11.1 | 33.8 |
| Avery | 21.5 (0.9) | 21.4 (1) | 9.4 | 32.5 |
| Beaufort | 28.3 (1.1) | 28.3 (1.5) | 17.7 | 38.0 |
| Bertie | 28 (1.2) | 28 (1.5) | 17.3 | 38.3 |
| Bladen | 28.6 (1) | 28.5 (1.2) | 17.8 | 39.3 |
| Brunswick | 28.5 (1) | 28.5 (1.4) | 18.3 | 38.1 |
| Buncombe | 23.6 (0.8) | 23.5 (1) | 14.1 | 35.2 |
| Burke | 26.1 (0.9) | 26.1 (1) | 14.9 | 37.6 |
| Cabarrus | 27.9 (1) | 27.8 (1.3) | 17.0 | 39.6 |
| Caldwell | 26 (0.9) | 26 (1.1) | 14.6 | 37.7 |
| Camden | 28 (1.2) | 27.9 (1.5) | 18.8 | 37.2 |
| Carteret | 28.3 (0.9) | 28.2 (1.3) | 19.3 | 37.1 |
| Caswell | 27.5 (1) | 27.4 (1.3) | 16.1 | 38.7 |
| Catawba | 27.1 (0.9) | 27 (1) | 15.6 | 38.2 |
| Chatham | 28 (1) | 27.9 (1.3) | 17.4 | 39.3 |
| Cherokee | 24.6 (0.9) | 24.5 (1.1) | 11.5 | 37.5 |
| Chowan | 28.1 (1.2) | 28.1 (1.4) | 18.3 | 37.4 |
| Clay | 23.6 (0.8) | 23.5 (0.9) | 13.7 | 35.9 |
| Cleveland | 27.2 (0.9) | 27.1 (1) | 15.1 | 38.7 |
| Columbus | 28.5 (1) | 28.5 (1.3) | 17.7 | 39.3 |
| Craven | 28.2 (1.1) | 28.2 (1.4) | 17.8 | 37.6 |
| Cumberland | 28.7 (1) | 28.6 (1.3) | 17.8 | 39.7 |
| Currituck | 28.1 (1.2) | 28 (1.5) | 19.6 | 36.8 |
| Dare | 28.2 (1.1) | 28.2 (1.3) | 18.9 | 36.3 |
| Davidson | 27.3 (0.9) | 27.2 (1.2) | 17.0 | 38.5 |
| Davie | 27.1 (0.9) | 27 (1.1) | 16.4 | 38.4 |
| Duplin | 28.2 (1) | 28.2 (1.4) | 16.6 | 38.5 |
| Durham | 27.8 (1.1) | 27.7 (1.4) | 16.0 | 39.2 |
| Edgecombe | 28.1 (1.1) | 28 (1.4) | 17.2 | 39.1 |
| Forsyth | 27.1 (1) | 27 (1.1) | 16.6 | 38.3 |
| Franklin | 27.8 (1.1) | 27.7 (1.4) | 15.7 | 39.0 |
| Gaston | 27.7 (0.9) | 27.6 (1.2) | 15.8 | 38.9 |
| Gates | 27.9 (1.1) | 27.8 (1.4) | 18.0 | 38.3 |
| Graham | 23.4 (0.9) | 23.3 (1.1) | 10.3 | 35.7 |
| Granville | 27.6 (1) | 27.5 (1.4) | 15.6 | 38.8 |
| Greene | 28.4 (1.1) | 28.3 (1.5) | 16.6 | 38.9 |
| Guilford | 27.4 (1) | 27.3 (1.2) | 17.2 | 38.4 |
| Halifax | 28 (1.1) | 27.9 (1.4) | 17.2 | 39.5 |
| Harnett | 28.4 (1) | 28.3 (1.3) | 17.1 | 39.5 |
| Haywood | 21.5 (0.8) | 21.4 (1) | 9.2 | 33.0 |
| Henderson | 24.3 (0.8) | 24.2 (1) | 15.0 | 35.4 |
| Hertford | 27.9 (1.2) | 27.9 (1.4) | 17.7 | 38.7 |
| Hoke | 28.5 (0.9) | 28.4 (1.2) | 17.9 | 40.2 |
| Hyde | 28.4 (1) | 28.4 (1.3) | 20.0 | 37.1 |
| Iredell | 26.9 (0.9) | 26.9 (1.1) | 15.6 | 38.2 |
| Jackson | 22.2 (0.8) | 22.1 (1) | 9.5 | 33.9 |
| Johnston | 28.4 (1) | 28.3 (1.3) | 16.1 | 39.1 |
| Jones | 28.1 (1.1) | 28.1 (1.5) | 17.0 | 38.2 |
| Lee | 28.1 (1) | 28 (1.3) | 17.3 | 39.5 |
| Lenoir | 28.3 (1.1) | 28.3 (1.5) | 16.8 | 38.4 |
| Lincoln | 27.3 (0.9) | 27.3 (1.1) | 15.5 | 38.4 |
| McDowell | 25.4 (0.9) | 25.3 (1) | 14.4 | 37.7 |
| Macon | 22.8 (0.8) | 22.7 (0.9) | 9.9 | 34.7 |
| Madison | 24.1 (0.8) | 24.1 (1) | 13.8 | 36.6 |
| Martin | 28.2 (1.1) | 28.1 (1.4) | 16.8 | 38.3 |
| Mecklenburg | 28 (0.9) | 27.9 (1.2) | 16.7 | 39.6 |
| Mitchell | 22.2 (0.9) | 22.2 (1) | 9.7 | 34.1 |
| Montgomery | 28.1 (0.9) | 28 (1.2) | 17.9 | 40.1 |
| Moore | 28.1 (1) | 28.1 (1.2) | 18.1 | 39.9 |
| Nash | 27.9 (1.1) | 27.8 (1.4) | 16.0 | 39.2 |
| New Hanover | 28.7 (1) | 28.6 (1.3) | 18.2 | 38.1 |
| Northampton | 28 (1.1) | 27.9 (1.4) | 17.3 | 39.4 |
| Onslow | 28.2 (1) | 28.1 (1.3) | 17.5 | 37.7 |
| Orange | 27.5 (1) | 27.4 (1.3) | 16.4 | 38.8 |
| Pamlico | 28.4 (1) | 28.4 (1.3) | 18.9 | 37.4 |
| Pasquotank | 28 (1.2) | 28 (1.4) | 18.7 | 37.2 |
| Pender | 28.4 (1) | 28.3 (1.3) | 17.2 | 38.5 |
| Perquimans | 28.1 (1.2) | 28 (1.4) | 18.4 | 37.4 |
| Person | 27.2 (1) | 27.1 (1.4) | 15.3 | 38.5 |
| Pitt | 28.3 (1.2) | 28.4 (1.5) | 16.6 | 38.6 |
| Polk | 26.9 (0.9) | 26.8 (1.2) | 16.3 | 39.1 |
| Randolph | 27.7 (1) | 27.6 (1.3) | 17.5 | 39.0 |
| Richmond | 28.5 (0.9) | 28.4 (1.3) | 18.5 | 40.7 |
| Robeson | 28.7 (0.9) | 28.7 (1.2) | 17.7 | 39.9 |
| Rockingham | 27.4 (1) | 27.3 (1.2) | 16.5 | 38.5 |
| Rowan | 27.3 (0.9) | 27.3 (1.2) | 16.9 | 38.8 |
| Rutherford | 27 (0.9) | 26.9 (1) | 15.5 | 39.0 |
| Sampson | 28.4 (1) | 28.3 (1.3) | 16.9 | 38.7 |
| Scotland | 28.6 (0.9) | 28.6 (1.2) | 17.9 | 40.5 |
| Stanly | 28.2 (0.9) | 28.1 (1.3) | 17.9 | 40.2 |
| Stokes | 26.7 (1) | 26.7 (1.2) | 15.8 | 38.2 |
| Surry | 26.1 (0.9) | 26 (1.1) | 14.3 | 38.5 |
| Swain | 22.1 (0.8) | 22.1 (1.1) | 9.3 | 34.2 |
| Transylvania | 23.1 (0.8) | 23 (0.9) | 14.2 | 34.1 |
| Tyrrell | 28.3 (1.1) | 28.3 (1.4) | 18.3 | 37.3 |
| Union | 28.3 (0.9) | 28.1 (1.2) | 17.6 | 40.3 |
| Vance | 27.7 (1.1) | 27.5 (1.4) | 15.9 | 39.0 |
| Wake | 28.1 (1) | 28 (1.4) | 16.4 | 39.1 |
| Warren | 27.8 (1.2) | 27.8 (1.5) | 16.5 | 39.5 |
| Washington | 28.2 (1.1) | 28.1 (1.5) | 17.5 | 37.8 |
| Watauga | 22.1 (0.9) | 22 (1) | 12.3 | 33.2 |
| Wayne | 28.4 (1.1) | 28.3 (1.4) | 16.6 | 38.8 |
| Wilkes | 25.9 (0.9) | 25.9 (1) | 13.9 | 38.0 |
| Wilson | 28.3 (1.1) | 28.2 (1.4) | 16.4 | 39.1 |
| Yadkin | 26.7 (0.9) | 26.6 (1.1) | 15.4 | 38.4 |
| Yancey | 22 (0.9) | 21.9 (1) | 9.6 | 33.9 |

S2 Table . Average statistics of heatwave days for all counties in North Carolina between 2008 and 2021.
