# Supplementary material for: Assessing the impact of heatwaves on emergency visits for major depression and suicidal ideation in youth with attention-deficit/hyperactivity disorder
Source: PLOS Ment Health. 2025 Oct 29;2(10):e0000444. doi: 10.1371/journal.pmen.0000444 (PMC12798237; doi:10.1371/journal.pmen.0000444)
Supplement: S3 Table — Poisson mixed-effect regression models for same-day, lagged, and cumulative effects were analyzed for each outcome. Models were also run to assess effect modification across covariates. (DOCX) [file pmen.0000444.s006.docx]

|  | **MDD** | | | **Suicide** | | | **Overlap** | | |
| --- | --- | --- | --- | --- | --- | --- | --- | --- | --- |
| **Exposure** | **RR** | **95% CI** | **p** | **RR** | **95% CI** | **p** | **RR** | **95% CI** | **p** |
| Same Day | 1.17 | (1.01, 1.34) | 0.04 | 1.07 | (0.99, 1.16) | 0.08 | 1.25 | (0.98, 1.60) | 0.08 |
| Lag 1 | 1.17 | (1.01, 1.35) | 0.03 | 1.08 | (1.00, 1.17) | 0.05 | 1.27 | (1.00, 1.62) | 0.05 |
| Lag 2 | 1.14 | (0.99, 1.32) | 0.07 | 1.03 | (0.96, 1.12) | 0.41 | 1.23 | (0.96, 1.57) | 0.41 |
| Lag 3 | 1.09 | (0.95, 1.27) | 0.23 | 1.03 | (0.96, 1.12) | 0.41 | 1.14 | - | 0.40 |
| Lag 4 | 1.02 | (0.88, 1.19) | 0.78 | 1.01 | (0.93, 1.09) | 0.84 | 1.06 | (0.82, 1.37) | 0.84 |
| Lag 5 | 1.00 | (0.86, 1.16) | 0.98 | 1.02 | (0.94, 1.11) | 0.63 | 1.01 | (0.77, 1.31) | 0.63 |
| Lag 6 | 0.98 | (0.85, 1.14) | 0.83 | 1.06 | (0.98, 1.14) | 0.18 | 0.97 | (0.75, 1.27) | 0.18 |
| Lag 7 | 0.95 | (0.82, 1.11) | 0.54 | 1.05 | (0.97, 1.14) | 0.23 | 1.01 | (0.78, 1.32) | 0.23 |
| 3-day cumulative | 1.14 | (1.06, 1.23) | 0.00 | 1.05 | (1.01, 1.10) | 0.01 | 1.22 | (1.08, 1.38) | 0.01 |
| 5-day cumulative | 1.10 | (1.04, 1.17) | 0.00 | 1.04 | (1.01, 1.08) | 0.01 | 1.17 | (1.05, 1.29) | 0.01 |
| 7-day cumulative | 1.07 | (1.01, 1.12) | 0.01 | 1.05 | (1.02, 1.08) | 0.00 | 1.12 | (1.03, 1.23) | 0.00 |
| Male | 1.21 | (0.98, 1.48) | 0.07 | 1.03 | (0.92, 1.14) | 0.63 | 1.17 | (0.81, 1.71) | 0.60 |
| Female | 1.13 | (0.93, 1.37) | 0.23 | 1.14 | (1.01, 1.28) | 0.04 | 1.31 | (0.95, 1.80) | 0.18 |
| Age 5-11 | 1.02 | (0.53, 1.97) | 0.95 | 1.01 | (0.82, 1.25) | 0.91 | 1.89 | (0.51, 7.00) | 0.90 |
| Age 12-17 | 1.19 | (0.98, 1.45) | 0.08 | 1.19 | (1.07, 1.34) | 0.00 | 1.43 | (1.02, 1.99) | 0.01 |
| Age 18-25 | 1.15 | (0.92, 1.43) | 0.21 | 0.96 | (0.84, 1.09) | 0.51 | 1.04 | (0.72, 1.51) | 0.53 |
| White | 1.25 | (1.05, 1.49) | 0.01 | 1.07 | (0.97, 1.19) | 0.17 | 1.25 | (0.94, 1.68) | 0.17 |
| Black | 0.97 | (0.73, 1.28) | 0.82 | 1.14 | (0.98, 1.33) | 0.08 | 1.17 | (0.71, 1.92) | 0.08 |
| Other | 0.87 | (0.46, 1.62) | 0.65 | 0.87 | (0.46, 1.62) | 0.65 | 1.34 | (0.38, 4.74) | 0.67 |
| Hyperactive | 0.70 | (0.49, 1.00) | 0.05 | 0.94 | (0.81, 1.09) | 0.43 | 0.46 | (0.23, 0.94) | 0.43 |
| Inattentive | 1.50 | (0.95, 2.36) | 0.08 | 0.88 | (0.64, 1.22) | 0.44 | 2.70 | (1.35, 5.39) | 0.43 |
| Combined | 1.46 | (1.02, 2.08) | 0.04 | 1.01 | - | - | 1.27 | (0.64, 2.50) | - |
| Unspecified | 1.24 | (1.03, 1.49) | <0.00 | 1.18 | - | - | 1.37 | (1.00, 1.86) | - |
| Hispanic | 0.90 | (0.44, 1.83) | 0.76 | 1.06 | (0.71, 1.57) | 0.76 | 1.86 | (0.43, 8.12) | 0.80 |
| Non-Hispanic | 1.18 | (1.02, 1.37) | 0.03 | 1.05 | (0.97, 1.14) | 0.07 | 1.20 | (0.93, 1.54) | 0.23 |

- Indicates that model could not compute RR or 95% confidence interval.

S3 Table. Relative Risk (RR), 95% Confidence Intervals (95% CI), and p-value (p) from the sensitivity analysis, where the analysis was limited to cases occurring in the warm season only. Poisson mixed-effect regression models for same-day, lagged, and cumulative effects were analyzed for each outcome. Models were also run to assess effect modification across covariate
