# Supplementary material for: Assessing the impact of heatwaves on emergency visits for major depression and suicidal ideation in youth with attention-deficit/hyperactivity disorder
Source: PLOS Ment Health. 2025 Oct 29;2(10):e0000444. doi: 10.1371/journal.pmen.0000444 (PMC12798237; doi:10.1371/journal.pmen.0000444)
Supplement: S4 Table — Poisson mixed-effect regression models were run for same-day, lagged, and cumulative effects. Models were also run to assess effect modification across covariates. (DOCX) [file pmen.0000444.s007.docx]

|  | **ADHD** | | |
| --- | --- | --- | --- |
| **Exposure** | **RR** | **95% CI** | **p** |
| Same Day | 0.98 | (0.97, 1.00) | 0.11 |
| Lag 1 | 0.98 | (0.97, 1.00) | 0.15 |
| Lag 2 | 0.98 | (0.97, 1.00) | 0.05 |
| Lag 3 | 0.98 | (0.97, 1.00) | 0.04 |
| Lag 4 | 0.98 | (0.97, 1.00) | 0.04 |
| Lag 5 | 0.99 | (0.98, 1.01) | 0.41 |
| Lag 6 | 0.99 | (0.98, 1.01) | 0.63 |
| Lag 7 | 0.98 | (0.97, 1.01) | 0.19 |
| 3-day cumulative | 0.98 | (0.98, 0.99) | 0.00 |
| 5-day cumulative | 0.98 | (0.98, 0.99) | 0.00 |
| 7-day cumulative | 0.98 | (0.98, 0.99) | 0.00 |
| Male | 0.98 | (0.96, 1.00) | 0.08 |
| Female | 0.99 | (0.97, 1.02) | 0.64 |
| Age 5-11 | 0.97 | (0.95, 1.00) | 0.12 |
| Age 12-17 | 0.98 | (0.96, 1.01) | 0.29 |
| Age 18-25 | 1.00 | (0.97, 1.03) | 0.89 |
| White | 0.97 | (0.96, 1.00) | 0.04 |
| Black | 1.01 | (0.98, 1.04) | 0.65 |
| Other | 0.96 | (0.63, 1.46) | 0.08 |
| Hyperactive | 0.97 | (0.95, 1.00) | 0.08 |
| Inattentive | 0.91 | (0.87, 0.97) | 0.00 |
| Combined | 0.98 | - | - |
| Unspecified | 1.01 | - | - |
| Hispanic | 1.66 | (0.80, 3.43) | 0.76 |
| Non-Hispanic | 1.04 | - | - |

- indicates that model could not compute RR or 95% confidence interval.

S4 Table. Relative Risk (RR), 95% Confidence Intervals (95% CI), and p-value (p) from the sensitivity analysis, examining ADHD-related ED visits defined by either a primary or secondary diagnosis of ADHD. Poisson mixed-effect regression models were run for same-day, lagged, and cumulative effects. Models were also run to assess effect modification across covariates.
